# Neutrophils in Tuberculosis-Associated Inflammation and Lung Pathology

**DOI:** 10.3389/fimmu.2020.00962

**Published:** 2020-05-27

**Authors:** Caleb N. Muefong, Jayne S. Sutherland

**Affiliations:** Vaccines and Immunity Theme, Medical Research Council Unit, The Gambia at the London School of Hygiene and Tropical Medicine, Fajara, The Gambia

**Keywords:** tuberculosis, neutrophils, inflammatory mediators, lung damage, sequelae

## Abstract

Protective immunity to *Mycobacterium tuberculosis (Mtb)*—the causative agent of tuberculosis (TB)—is not fully understood but involves immune responses within the pulmonary airways which can lead to exacerbated inflammation and immune pathology. In humans, this inflammation results in lung damage; the extent of which depends on specific host pro-inflammatory processes. Neutrophils, though increasingly linked to the development of inflammatory disorders, have been less well studied in relation to TB-induced lung pathology. Neutrophils mode of action and their specialized functions can be directly linked to TB-specific lung tissue damage observed on patient chest X-rays at diagnosis and contribute to long-term pulmonary sequelae. This review discusses aspects of neutrophil activity associated with active TB, including the resulting inflammation and pulmonary impairment. It highlights the significance of neutrophil function on TB disease outcome and underlines the necessity of monitoring neutrophil function for better assessment of the immune response and severity of lung pathology associated with TB. Finally, we propose that some MMPs, ROS, MPO, S100A8/A9 and Glutathione are neutrophil-related inflammatory mediators with promising potential as targets for developing host-directed therapies for TB.

## Introduction

Tuberculosis (TB) is the single deadliest infectious disease known to man with 10 million new cases and 1.6 million deaths (including 300,000 HIV coinfected) in 2018 (1). This report does not account for health impairment nor deaths during and following TB treatment; which is suggested to be about three times higher than those observed in the general population or suitably matched controls (2). It is known today that despite being diagnosed as microbiologically cured from TB, about 50% of patients still suffer from some form of pulmonary impairment after tuberculosis (PIAT), irrespective of smoking habits (3). The definition of PIAT encompasses several clinical conditions; which in former TB patients is suggested to result from chronic inflammation, characterized by disrupted pulmonary structure and function (residual lung deficits) (4, 5); a state described as thoracic/TB sequelae (6). These include parenchymal, airway, vascular and mediastinal lesions manifested mainly through structural [cicatrization, calcification, fibrosis and reduction in cavity wall thickness (6)] and functional [deficit in forced expiratory volume (4)] damage; the establishment (7) and severity (8–10) of which, are associated to neutrophil abundance and (hyper-)activity.

As compelling as these effects of TB may be, PIAT is presently not included in global estimates of TB burden despite increasing scientific interest and evidence of associated morbidity and mortality (3–5, 11–14).

A recent cohort study showed that subjects with a history of fully treated active TB (ATB) lost 3.6 years on average of disability-adjusted life expectancy compared to subjects with latent TB infection (LTBI) who did not progress to the active state (12). Despite the lack of data to support disease burden resulting from long-term sequelae (11), the above reduced life expectancy is a direct result of TB sequelae and suggests that a considerable proportion of the TB disease burden is contributed by subjects who have successfully cleared Mtb. Indeed, a study in Texas, USA reported that the number of years lived with chronic TB disability accounts for 75% of non-fatal health effects of TB (11). This same study suggests that the most health and financial savings may be achieved by preventing rather than shortening therapeutic strategies. Additionally, a recent retrospective study reveals the negative effect of drug-resistance and disease recurrence on PIAT (15). Consequently, early detection of parameters which increase the likelihood of ATB complication into chronic inflammation and long-term sequelae would inform clinicians on the need for case-specific treatment measures and contribute to minimizing the global TB burden (13). Such parameters can be realistically linked to neutrophil function and/or interaction with other immune cell populations in the view of their specific activities described in subsequent sections below.

Generally, protective immunity to *Mycobacterium tuberculosis* (Mtb)—the causative agent of TB—is a combination of innate and adaptive immune responses within the pulmonary airways via which this pathogen gains entrance into the human host (16, 17). This immune response to TB is described as a chronic granulomatous inflammation; caused by close interaction between Mtb bacilli and host immune agents at the infection site (18). Indeed, the term “chronic granulomatous” draws from a condition, chronic granulomatous disease (CGD), with similar inflammatory outcomes; resulting from genetic mutations of reduced nicotinamide adenine dinucleotide phosphate (NADPH_2_) oxidase-encoding genes (19, 20). Disruption in the production of this enzyme; which normally catalyzes the synthesis of reactive oxygen species (ROS) used by phagocytes to destroy bacteria during phagocytosis, leads to enhanced susceptibility to infectious pathogens and granuloma formation; particularly in the lungs (21). Despite several gaps in knowledge, the contribution of adaptive immune responses: particularly T-cells [reviewed in Jasenosky et al. (22)] and to a lesser extent B-cells [reviewed in Achkar et al. (23)] have been addressed. Furthermore Dyatlov et al. recently reviewed the role of B cells on reducing neutrophil influx to infection sites (24) and; these Mtb-specific immune responses having been studied extensively and will not form a focus of this review.

Recent studies have revealed that the innate arm of the immune system plays a bigger role in the onset and regulation of inflammatory processes during ATB than previously thought. ROS-generating cells are central to Mtb-induced inflammatory response; and that they are main actors of relevant cell death processes (i.e., apoptosis, necrosis, pyroptosis, necroptosis, pyronecrosis, NETosis, and autophagy) that influence TB disease progression [reviewed by Mohareer et al. (25)], suggests that their activity contributes considerably to destructive immunity to Mtb infection. The aim of this review is to provide an update on the importance of neutrophils during ATB and to identify related immune mediators associated with anti-TB treatment response and lung damage.

## TB-induced Inflammatory Response

Innate immune responses play a central role in the pathology of infectious and inflammatory diseases including acute abdominal inflammation (26), cancers (27, 28) and respiratory tract disorders (29, 30). Phagocytic cells (i.e., neutrophils and macrophages) are the predominant components of this response in TB (17). In collaboration with inflammatory mediators like cytokines (31) and proteases, they are key contributors to the host interaction with Mtb, in a process which generally ends with the destruction of the pathogen and resolution of inflammation (32). In many cases, however, the inflammatory response is relatively ineffective and can lead to destruction of host tissues as reviewed by Fullerton and Gilroy (33). Such an unwanted scenario is characterized by a constant influx of inflammatory mediators and innate immune cells to the site of infection with progressive deterioration of the affected tissue. The end result is the formation of tuberculous granulomas whose structure, immune/pathogen cell balance (34), and intrinsic T-cell activity (35) ultimately determine the degree of formation of tissue lesions (36).

### Defining and Assessing Lung Impairment

In order to understand the role of neutrophils in lung pathology, we need consensus on structural versus functional impairment. There are currently no international guidelines describing how to classify levels of structural impairment following TB as well as identifying TB sequelae in general (37). ATB is increasingly further classified with respect to disease severity into the extent of functional and/or structural lung damage, however, a decisive classification of TB patient pathology has not been reached at this time. Nonetheless, certain criteria have allowed the severity of active pulmonary TB to be determined following assessment of impaired pulmonary function via spirometry testing (38) and the observation of lesions and/or lung cavities through chest x-rays (CXR) and computed tomography (CT) (39).

Structural lung abnormalities determined by x-ray or computed topography (CT) scores have been observed to correlate to a degree with lung function in pulmonary TB (40). Reports also suggest that functional pulmonary impairment at diagnosis only begins to improve significantly several months after the end of successful TB therapy (4, 40). Saldana et al. observed that CXR abnormalities are inversely proportional to and more reliable than spirometry evaluations when assessing severity of lung impairment in cured ATB patients (41). An even earlier study by Plit et al. showed that the change in CXR score (pre- vs. post-treatment) is the most reliable predictor of the severity of functional lung impairment in ATB: here too, an inverse proportionality was observed between CXR scores and forced expiratory volume (FEV1; a spirometric parameter) (42). These suggest that monitoring variations in structural impairment during TB therapy is essential (or at least of significant added value) when attempting to determine the extent of TB sequelae. However, whilst most relevant clinical studies have generally attempted to monitor ATB-linked signs of TB sequelae via assessment of dyspnoea and disrupted lung function by spirometry (3, 4, 11, 14, 43–45), fewer cases have accounted for both structural and functional damage (13, 41, 42, 46), and none focussing on the former exclusively (see Table 1). Relevant follow-up parameters, where available (especially involving longitudinal cohort studies), appear to have relied on the researchers' study objective and understanding of TB sequelae—variably assessing different forms of pulmonary damage, lung rehabilitation and even treatment responses but not the potential inflammatory triggers of these events as the Ravimohan group's latest review hints (49). This is probably owing to absence of a referential guideline as mentioned above. At this time, a few studies: Ravimohan et al. (48) and Plit et al. (42) have assessed severity of lung impairment in ATB in relation to the expression of inflammatory mediators: matrix metalloproteinases (MMPs) in the former and; serum c-reactive protein (CRP), serum α1-protease inhibitor (α1-PI) and urine cotinine in the latter. To account for these limitations, a multisite trial is currently underway to monitor host-pathogen and socioeconomic factors that influence the development of pulmonary sequelae in ATB patients (50).

**Table 1:** Clinical studies assessing TB sequelae.

**References**	**Study site**	**Study type and design post-treatment commencement**	**ATB sample size, *n***	**Nature of residual lung impairment**	**Associated inflammatory response**
Ngahane et al. (46)	Cameroon	Cross-sectional Includes HIV^+^	269	Structural (CXR lesions) and Functional (dyspnoea and spirometry)	No
Ralph et al. (13)	Indonesia	Longitudinal [Baseline (BL), 6M & over 6M] Includes HIV^+^	200	Structural (CXR score of lesions and cavitation) and Functional (dyspnoea, SGRQ, spirometry)	No
Kumar et al. (47)	India	Longitudinal (BL & 6M) No HIV^+^ cases Part of larger study involving patients with co-morbidities	24	Structural (cavitation; no CXR-score)	Yes
Ravimohan et al. (48)	USA	Prospective (over 6M) All TB/HIV^+^	14	Functional (spirometry)	Yes (MMPs)
Pasipanodya et al. (11)	USA	Longitudinal (BL, 6M & over 6M) Includes HIV^+^	177	Functional (SGRQ and spirometry)	No
Plit et al. (42)	South Africa	Longitudinal (BL & 6M) Includes HIV^+^	76	Structural (CXR score of lung infiltrates) Functional (spirometry)	Yes (c-reactive protein (CRP) and serum α1-protease inhibitor (α1-PI)
Cole et al. (43)	South Africa	Cross-sectional Includes HIV^+^	55	Functional (SGRQ and spirometry)	No
Patil and Patil (44)	India	Longitudinal (6M, 9M, & 12M) No HIV^+^ cases	118	Functional (dyspnoea and spirometry)	No
Hnizdo et al. (4)	South Africa	Retrospective (BL-−375M) Includes HIV^+^	2,599	Functional (spirometry)	No
Maguire et al. (45)	Indonesia	Longitudinal (BL, 2M, & 6M) Includes HIV^+^	115	Functional (dyspnoea, SGRQ, spirometry)	No
Saldana et al. (41)	Mexico	Cross-sectional Includes HIV^+^	127	Functional (Spirometry) and Structural (CXR abnormalities)	No
Vecino et al. (14)	USA	Longitudinal (BL, 6M, & over 6M)	123	Functional (Spirometry)	No
Chushkin et al. (3)	Russia	Prospective (Over 12M) Undetermined HIV status	214	Functional (Spirometry)	No

## Evidence of Neutrophil Impact on Destructive TB Inflammation

### Neutrophilia and Hyperinflammation

Polymorphonuclear neutrophils are the most abundant type of white blood cells and play a central role in the immune response to bacterial pathogens (51). The protective activity of neutrophils in TB infection is observed during granuloma formation where mycobacteria are phagocytosed from infected macrophages by oxidative killing (52).

Previous work indicates that the levels of granulocytes (neutrophils and eosinophils) in circulation are higher in patients with ATB disease than those with latent TB infection (LTBI); with levels decreasing significantly following successful TB treatment (53). It has also been demonstrated that neutrophilia independently associates not only with increased risk of cavity formation and lung tissue damage (54), but also mortality in patients undergoing TB therapy (55), suggesting that the neutrophil count in tuberculosis positively correlates with bacillary load and disease outcome. Recently, Leem et al. (56) monitored inflammatory markers in TB patients and found that the neutrophil counts and neutrophil to lymphocyte ratios (NLR) were decreased following a 6-months anti-TB drug therapy compared to baseline. These results hint that inflammation might be resolved only following the 6-month treatment completion, suggesting that progress to chronic inflammation and development of pulmonary lesions is a silent process potentially mediated by secondary products of inflammatory response whose activity persist in tissue long after mycobacterial clearance.

Despite the lack of a consensus on neutrophil classification, varying attributes: granule content (cytotoxic species/enzyme concentration), density (low or normal density granulocytes), nuclear segmentation [banded or (hyper)-segmented], tumor suppressive/enhancing functions (N1/N2), to surface antigen expression [CD177 (7, 57, 58); CD16, CD62L and CD11b] and cytokine/chemokine secretion levels have been associated to disease and immunoregulation [reviewed in Hellebrekers et al. (59), Perobelli et al. (60), and Wang (61)]. It is therefore arguable that a combination of these attributes could constitute a neutrophil profile suggestive of disease severity at an early stage as well as anticipated development of sequalae if chronic conditions (in TB potentially) were to be established. However, given the vast discrepancies in markers, experimental conditions and disease models investigated by previous studies as described in the reviews cited above, these functional differences in neutrophil subsets will not constitute a focus here. Nevertheless, the severity of ATB is linked to neutrophilia as discussed above; but also a specific hyperactivated profile of the circulating neutrophils; which has predominantly been associated with immature banded (or non-segmented) neutrophils (8). Interestingly, the bulk of neutrophil cytotoxic (and antibacterial) molecules are concentrated in their granules. Hence, neutrophil degranulation and exocytosis: processes requiring phosphatidylinositol 3-kinase, (PI3-K) (62); are closely related to the severity of neutrophil-mediated inflammation. We therefore anticipate that a potential neutrophil bio-signature of ATB would encompass enhancement/inhibition of some specific chemokines and increased neutrophil-specific enzyme concentrations. In fact, a recent review by Leisching (7) exposes the regulatory role of PI3-K on enhanced neutrophil mobility and hyperactivity and; the effect on neutrophil-driven TB inflammation. This hyperactivity is equally suggested to be at play in chronic periodontis where it is associated with increased migratory capacity as well as pro-inflammatory cytokine (IL-8, TNF, and IL-1, notably) production by circulating neutrophils (63). Taken together, neutrophil relative abundance (in circulation and at infection sites) and cytokine/enzyme release are potentially major agents of hyperinflammatory conditions observed in ATB.

The mechanisms responsible for this inflammatory response mainly result from three neutrophil functions: oxidative burst, necrosis and NETosis.

### Oxidative Burst Capacity

Although neutrophils have the capacity to protect against Mtb infection, if left uncontrolled their collective activity may produce pathogenic effects through different functions (64). One such phagocytic function is oxidative burst, which is the release of reactive oxygen species (ROS) mainly by neutrophils and to a lesser extent, macrophages during phagocytosis, a process which is mediated by nicotinamide adenine dinucleotide phosphate (NADP) oxidase (65). This antibacterial activity is performed by a myeloperoxidase system composed mainly of reduced NADP (NADPH_2_), reduced glutathione (GSH), azide, cyanide, thiocyanate, Tapazole, thiourea, cysteine, ergothioneine, thiosulfate, reduced nicotinamide adenine dinucleotide (NADH_2_), and tyrosine (66).

GSH levels have been shown to reduce significantly in PMBCs and red blood cells isolated from tuberculosis patients compared to healthy controls (67), while increased GSH levels are reported to enhance T-cell capacity to inhibit Mtb growth inside macrophages (68). Also, ROS produced by neutrophils during oxidative burst have been reported to drive Mtb-induced necrosis; which in turn promotes Mtb growth (69). It has also been suggested that rapid assessment of individual neutrophil oxidative burst capacity could distinguish patients at risk of excessive immune responses and thus could potentially guide therapy (70). Hence, correlating neutrophil oxidative burst capacity with GSH and/or NADPH_2_ levels in TB patients may provide avenues for novel host-directed therapies.

### Neutrophil Extracellular Traps (NETs)

*In-vitro* studies by Brinkmann (71) revealed that neutrophil activation with lipopolysaccharide (LPS), interleukin 8 or phorbol myristate acetate (PMA) led to the release of cell components, which form an extracellular fibril matrix called neutrophil extracellular traps (NETs). These components are proteins [namely neutrophil elastase (NE) and myeloperoxidase (MPO)], DNA and chromatin-derived fibers; which destroy bacteria extracellularly (71, 72). This process, NETosis, is a powerful neutrophil-mediated response to a range of infections but also acts as a double-edged sword during inflammatory diseases (73). Interestingly, neutrophils can sense pathogen size and can produce more NETs in presence of larger pathogens like *Mycobacterium bovis* (74). Although aggregated NETs are reported to degrade neutrophil-derived inflammatory mediators in an attempt to resolve inflammation (75), NETs also stimulate unwanted immune reactions and trigger tissue injury (73, 76).

In TB pathogenesis, Mtb is reported to induce the formation of NETs, which trap Mycobacteria *in vitro* but are unable to kill them (77). This may be partially explained by the fact that expression of enzyme systems such as those required in inflammatory pathways [i.e., to degrade proteins within the phagolysosome (e.g., MPO) and for the phagocytic burst, NADPH-oxidase complex and the generation of ROS] are suppressed (78). Furthermore, these Mtb-induced NETs are also associated with macrophage activation in humans (79) and could thus help elucidate specific inflammatory mechanisms of lung damage in TB pathogenesis. Indeed, a recent study by De Melo et al. revealed high levels of citrullinated H3—a common NET marker—in serum samples from TB patients with extensive pulmonary damage (54). Although this marker is usually measured in combination with others (i.e., MPO and NE) to specifically identify NETs, this study suggests that NET formation is centrally linked with severe lung tissue damage in TB patients and could be implicated in subsequent pulmonary pathology.

Neutrophil-mediated lung injury is not just restricted to Tuberculosis. For example, excessive neutrophil recruitment and NETosis was linked to acute lung injury in a mouse model of Influenza pneumonitis (80). Additionally, more recent studies reveal that reduced neutrophil recruitment into infected tissue promotes resolution of inflammation (81). Hence, monitoring NETosis and neutrophil-associated inflammatory mediators within inflamed tissue could be useful in developing therapeutic targets against chronic inflammatory conditions like TB (72).

### Metalloproteinases in Destructive TB Immunity

A group of molecules increasingly associated with excessive lung inflammation is the matrix metalloproteinases (MMPs). In the case of cystic fibrosis, which results in loss of pulmonary architecture, Pardo et al. described in a review (82) the essential role played by MMPs in modifying the tissue microenvironment and modulating cell signaling through their ability to degrade constituents of the extracellular matrix. Although the origin of most MMPs cannot be directly linked to neutrophils, MMP-9 is known to be secreted rapidly by neutrophils in whole blood from healthy volunteers following proinflammatory stimulus (83) and is suggested to facilitate transmembrane neutrophil migration (84); also reviewed in Pardo et al. (82). Similar to MMP-9, MMP-8 synthesis in ATB patients is also suspected to be of neutrophil origin (85). In effect, Ravimohan et al. (48) assessed the role of MMPs on TB-immune reconstitution inflammatory syndrome and observed increased MMP-8; whilst MMP-2, -3 and -9 levels reduced (MMP-1 did not vary significantly) in patients with impaired lung function post-TB cure following antiretroviral therapy. MMP-1 and MMP-8 have previously been shown to correlate with pulmonary tissue damage (PTD) in patients with ATB (85, 86) while MMP-14 has been shown to play a central role in TB pathogenesis by provoking collagen degradation and regulating monocyte migration (87). Interestingly, a more recent study by De Melo et al. found lower levels of serum MMP-8 in TB patients with severe PTD showing no radiological improvement after 60 days of anti-TB treatment (54). Whether this change in trend is related to plasma vs serum or due to the presence/absence of HIV infection is unknown, however, the latter observation is inconsistent with that from the previous studies reported above and suggests that MMP regulation in TB patients might differ between the circulation and the lung as well as in the presence of coinfection. Nonetheless, de Melo et al. (54) did find higher MMP-1 levels in these patients compared to those with improved chest-x rays. Hence, whilst there is clearly a role for MMPs in TB-linked tissue damage, more detailed studies, with assessment of coinfections, are required to ascertain which MMPs are predominant mediators. This will help to determine potential host-directed therapeutic strategies.

Furthermore, a few clinical studies on major TB comorbidities have recently emerged. One shows that sputum levels of MMP-1, -2, -3 and -9 are higher in HIV negative TB patients than in TB healthy controls (HC) and HIV positive TB patients with a correlation found between the degree of chest x-ray inflammation and both MMP-1 and MMP-3 levels in HIV negative TB patients (88). Moreover, the clinical severity of TB is known to increase in TB patients with diabetes mellitus (DM). Kumar et al. have shown that circulatory levels of MMP-1, -2, -3, -8, and -13 in these patients decrease following successful TB treatment and that MMP-1 (in sputum) and MMP-1, -2, -3, -9, and -12 levels (in serum) were higher in patients with more severe structural lung damage at baseline (47) as determined from chest x-rays.

These findings suggest that MMPs (MMP-1, -2, -3, -8, and -9, particularly) may promote tissue injury following Mtb infection. Hence, monitoring the correlation of these particular MMPs together with the downregulation of other neutrophil-related inflammatory proteins and pro-inflammatory cytokines associated with intracellular killing pathways during TB infection would increase our understanding of the active inflammatory pathways which enhance susceptibility to development of PIAT and subsequent sequelae. Importantly, natural regulation of MMP activity is performed by tissue inhibitors of metalloproteinases (TIMPs). The levels of TIMPs have not yet been monitored in TB patients; an aspect of TB research which should be addressed for optimal understanding of inflammatory mechanisms involved in development and host control of TB-related PTD.

## Neutrophil-related TB HDT

With increasing cases of co-infections, co-morbidities, drug resistance; as well as the cost associated with the relatively long standard antibiotic TB-treatment, new treatment regiments like host directed therapies (HDT) could complement existing Mtb-targeted approaches. Meanwhile biomarkers for efficiently identifying and treating TB disease progressors at an early stage are being actively researched (89), those that could single out individuals who develop unresolving inflammation-induced lung damage are still greatly under-investigated. This means that research on HDTs should ideally focus on diagnosis and prevention of the latter long-lasting condition as well. Recent reviews have highlighted various established as well as promising host directed adjuvant therapies against the development of TB disease (90), TB-linked inflammation (91, 92) and lung damage (93). Drugs that potentially inhibit pulmonary damage and/or promote lung repair range from steroids to nonsteroidal anti-inflammatory drugs, statins, metformin, dietary supplements, TNF blockers etc. (10). Of these, we observe that those suppressing pro-inflammatory aspects of the disease appear to be potent targets in preclinical and clinical trials. In fact, Young et al. have recently reviewed current targets in TB HDT with some of the most advanced ATB-relevant in clinical trials being modulators of pro-inflammatory mediators which: dampen inflammatory responses, curb immunopathology and resolve lung damage (93). These include the phase 3 drugs: cox-2 inhibitor (Meloxicam) and corticosteroids (Prednisolone and Dexamethasone) amongst others. This HDT potential of inflammatory mediators has also been addressed with inhibitory effects on neutrophil recruitment (Ibuprofen) and neutrophil-derived inflammatory mediators such as ROS and MPO as reviewed by Dallenga et al. (94).

Also, a combination of the anti-inflammatory drug, zileuton (an inhibitor of the synthesis of pro-inflammatory eicosanoid; already approved against asthma) and prostaglandin E2 (95) is reported to reduce bacillary load and TB-induced lung damage in mice. Statins are also interesting HDT targets against destructive lung pathology following ATB (96); with a promising phase 2 trial using pravastatin being investigated in South Africa (ClinicalTrials.gov Identifier: NCT03456102). Moreover, a phase 2b trial testing the effect of atorvastatin against PIAT in patients with or without HIV is about to begin in South Africa (ClinicalTrials.gov Identifier: NCT04147286); underlining the potential of these agents.

Potentially, some mediators of neutrophil function (mentioned in previous sections) could provide suitable HDT targets. Amongst others, these involve: vitamin D which is reported to inhibit Mtb-induced expression of MMP-7 and−10 as well as MMP-9 gene expression, secretion and activity by peripheral blood mononuclear cells (PBMCs) (97). Although the authors reported that the latter inhibition occurs irrespective of infection, MMP-9 is reported to be of neutrophil origin and in-depth investigation may be warranted. Doxycycline is also a known MMP-inhibitor which in TB-HIV co-infection particularly, is shown to suppress the secretion of TNF, MMP-1 and−9 by primary human macrophages while reducing Mtb growth in the guinea pig model of TB (98). Also, Allen et al. reviewed the importance of considering GSH as HDT against TB and TB/HIV co-infection (99). Furthermore, it is important to note that N-acetylated proline glycine proline (ac-PGP) induces neutrophil chemotaxis and neutrophil production of MMP-9 and IL-8 (100, 101) which has led this molecule to be suggested as potential HDT-target against chronic neuroinflammatory diseases (102) and cystic fibrosis (103); which result in MMP activation and result in considerable tissue damage like TB.

Besides these, calprotectin, a hetero-dimer made up of proteins S100A8 and S100A9 is a mediator of inflammatory responses and a potent diagnostic and HDT target against inflammatory diseases (104). Actually, Gopal et al. (9) reported that S100A8/A9 accumulates in TB-induced granulomas. The authors showed that this accumulation was neutrophil-driven (in humans) and the animal models they employed suggested an association between S100A8/A9 and the degree of inflammation and lung pathology during ATB. Recently, it has been shown that these high levels of S100A8/A9 as well as an S100A8/A9-mediated enhanced accumulation of neutrophils in lungs of mice and macaques are associated with Mtb proliferation in chronic TB disease (105). Both studies reveal a close interaction between neutrophils and S100A8/A9 in ATB suggesting that neutrophils and S100A8/A9, particularly could be targeted in TB HDT.

## Concluding Remarks and Future Perspectives

In conclusion, we believe that observation and monitoring of neutrophil subsets and related inflammatory mediators is important not only for studies aiming at developing novel therapeutic targets against TB (72) but also for improved estimation of host immuno-modulatory effects on the severity of TB sequalae. It is foreseeable that the extent of long-term pulmonary injury sustained and potentially resorbed following TB therapy (irrespective of HIV coinfection) could be correlated to a specific neutrophil function. It is also likely that ATB patients who express a specific form of neutrophil-mediated inflammatory response over the period from diagnosis through treatment are more susceptible to developing chronic PIAT than otherwise. A major challenge will be harmonizing the categorization of disease severity (structural and functional) to ease comparison between clinical studies. Moreover, we believe that prediction of treatment response and residual pulmonary impairment in future clinical studies would be made more effective and reproduceable by evaluating inflammatory responses as well as simultaneously monitoring variations in pulmonary structure and function during and months after treatment completion. Finally, prospective HDTs; which rely on inflammatory mediators of neutrophil activity particularly, should be investigated further.

## Author Contributions

CM was the primary author of the manuscript. JS provided substantial writing support, edits, and suggestions.

## Conflict of Interest

The authors declare that the research was conducted in the absence of any commercial or financial relationships that could be construed as a potential conflict of interest.
